# Cortical thinning with increasing OSA severity stages in adult comorbid insomnia and sleep apnea and its relevance for respiration measures

**DOI:** 10.1038/s41598-026-55300-7

**Published:** 2026-05-28

**Authors:** Liping Pan, Lifeng Hang, Hui Li, Yi Yin, Liming Li, Wenfeng Zhan, Yongli Li, Quanxin He, Jingping Yang, Huiyu Chen, Yuting Wu, Shujuan Qiu, Shumei Li, Guihua Jiang

**Affiliations:** 1 The Affiliated Guangdong Second Provincial General Hospital of Jinan University, Guangzhou, China; 2 Heyuan People’s Hospital, Heyuan, China; 3 Department of Medical Imaging, Guangdong Second Provincial General Hospital, Guangzhou, China; 4 People’s Hospital of Xinjiang Uygur Autonomous Region, Urumqi, Xinjiang China; 5 Department of Health Management, Henan Provincial People’s Hospital, Zhengzhou University People’s Hospital, Henan, China; 6 Xiamen Humanity Hospital Fujian Medical University, Fujian, China

**Keywords:** Comorbid insomnia and sleep apnea, Cortical thickness, Obstructive sleep apnea, Insomnia disorder, Hypoxia, Supramarginal gyrus, Diseases, Medical research, Neurology, Neuroscience

## Abstract

The study aimed to analyze the alterations of cortical thickness (CT) in comorbidity of insomnia and sleep apnea (COMISA) patients of varying severity and the relationships of these CT changes with clinical measures. The study included 34 COMISA patients and 24 healthy controls (HCs). The patients received structural MRI as well as measures associated with respiration, sleep, cognition, and mood, and were classified as mild and moderate (MC) or severe (SC) groups. The CT for COMISA was analyzed through surface-based morphometry (SBM). Voxel-wise differences in CT between two groups were analyzed. Individual CT maps of MC, SC, and HC groups were used in analysis of variance for investigating how COMISA severity at various stages affects CT. Partial correlation analysis was used for analyzing the relations between changes in CT and clinical measures. Reduced CT was located in four clusters, including left supramarginal gyrus, left precentral and postcentral gyri, right pericalcarine and lingual gyri, and right rostral and caudal middle frontal gyri in COMISA group. The CT of left supramarginal gyrus was found in MC group, whereas all four clusters in SC group Moreover, the partial correlation analysis results revealed that CT was significantly related to respiration measures (AHI and LSaO_2_). In conclusion, our findings showed a greater cortical thinning with increasing OSA severity within the COMISA cohort, which was negatively correlated with respiration measures, suggesting that respiratory impairment (e.g., intermittent hypoxia) may contribute to the neuroanatomical alterations in these patients.

**Supplementary Information:**

The online version contains supplementary material available at 10.1038/s41598-026-55300-7.

## Introduction

Insomnia disorder (ID) and obstructive sleep apnea (OSA) are sleep diseases with the highest prevalence rates and significantly contribute to cognitive impairment, mood disorders, and daytime fatigue^1–3^. OSA exhibits the typical features of snoring and choking while sleeping at night, resulting in cortical arousal, hypopnea, apnea, and the declined blood oxygen saturation^4,5^. On the other hand, ID is associated with the difficulty in falling or being asleep and waking up early in the morning. OSA and ID often coexist, and the comorbidity of insomnia and sleep apnea (COMISA) may occur in 30–50% of patients^6^.A previous study reported that COMISA had a higher incidence than ID or OSA^7^. Additionally, individuals with COMISA experience a greater incidence of cognitive and behavioral impairments^8^ and psychiatric disorders than those with only ID or OSA^8,9^. Although COMISA has significant cognitive and mood effects, our understanding of the neuroanatomical alterations associated with this comorbidity is limited.

Several studies have investigated cortical thickness (CT) in patients suffering from OSA and ID by conducting high-resolution magnetic resonance imaging (MRI). Some brain regions associated with OSA, including the precentral and postcentral gyri, superior temporal gyrus, left dorsal posterior insula, and right inferior parietal gyrus, show CT alterations^10–13^. Researchers have postulated that a decrease in CT might signify atrophy caused by long-term damage, such as the loss of neurons and supporting glial cells due to repeated intermittent hypoxic episodes^13,14^, leading to working memory deficits, motor dysfunction, or reduction in speech encoding^15,16^. Moreover, Chokesuwattanaskul et al. ^17^ classified participants into mild or severe hypoxia based on hypoxia parameters, such as the lowest oxygen saturation, oxygen desaturation index, and total sleep time < 90% oxygen saturation, and reported cortical thinning in right inferior parietal and frontal gyri of severe desaturation group as well as in superior parietal gyrus of high oxygen desaturation index group. When considering patients with ID, however, a degree of inconsistency was found in CT changes. CT was found to increase and decrease among ID patients^18,19^.

Although several structural neuroimaging studies have investigated changes in CT in individuals with ID or OSA alone, CT changes specific to COMISA are limited. We note that the same cohort has been previously used to examine gray matter volume (GMV) alterations in COMISA^20^; the present study extends this work by focusing on cortical thickness (CT), a distinct morphometric measure that provides different biological information. A growing body of literature indicates that CT and GMV reflect complementary aspects of brain morphology: genetic studies have shown that cortical thickness and surface area are genetically independent^21^, methodologically CT is less confounded by head size than GMV^22^, and disease-based studies have demonstrated that these two metrics exhibit distinct regional vulnerability patterns^23^. Thus, analyzing CT in the same cohort can yield novel insights into COMISA-related brain pathology that are not reducible to previous GMV findings. Therefore, our current study used surface-based morphometry (SBM) to compute whole-brain cortical thickness (CT) at the voxel-wise level and compared CT alterations between patients with COMISA and healthy controls (HCs), all of whom completed measures of respiration, sleep, cognition, and mood. Additionally, we further explored the CT alterations at different severity stages of patients with COMISA and the associations of respiration, sleep, cognition, and mood measures.

## Materials and methods

### Subjects

The ethics committee of People’s Hospital of Xinjiang Uygur Autonomous Region approved the present prospective study. The same participant cohort has been described previously in our gray matter volume study^20^. The present study reports a separate analysis of cortical thickness, which has not been previously published. All procedures were carried out in accordance with the principles outlined in the 1964 Declaration of Helsinki and its subsequent revisions, as well as in the relevant guidelines. Written informed consent was obtained from all participants. In total, 63 COMISA patients were enrolled at the People’s Hospital of Xinjiang Uygur Autonomous Region Provincial General Hospital as research participants. All enrolled COMISA cases were recently identified through diagnostic procedures and had not initiated any therapeutic regimens, including pharmacological treatments, for their condition prior to study participation. Patients below were included: (1) those meeting OSA diagnosis criteria in the International Classification of Sleep Disorders, Version 3 (ICSD-3)^24^; (2) patients who satisfied the criteria of ICSD-3 chronic insomnia disorder, i.e., insomnia symptoms appeared thrice or more weekly for more than three months; (3) all patients were right-handed; (4) all patients were 18–60 years old.

The diagnostic requirements for OSA in the ICSD-3 stipulate that patients must meet (A) and (B) or (C). In line with (A), the cases should exhibit the following symptoms: (1) related signs/symptoms, like drowsiness, insomnia, snoring, observed apnea, fatigue, and subjective nocturnal breathing disorder; (2) or a related medical or mental disease, such as hypertension, coronary artery disease, atrial fibrillation, congestive heart failure, diabetes, stroke, emotional disorder, or cognitive dysfunction. For (B), patients should display at least five major obstructive respiratory events, including hypopneas, obstructive and mixed apneas, or arousal associated with dyspnea, according to the American Academy of Sleep Medicine (AASM) scoring manual. Finally, under (C), at least 15 obstructive respiratory events should take place every hour, even if there are no associated symptoms or disorders. The exclusion criteria were as follows: (1) drug addiction or mental diseases; (2) nervous system disorders or unstable or severe somatic disorders; (3) suicidal ideation, severe self-injury, agitation, impulse injury, or conditions that are difficult or impossible to cooperate with inspection; (4) contraindications of MRI; and (5) excess head movement during MRI examinations. Prior to neuroimaging procedures, all enrolled participants received comprehensive overnight polysomnographic (PSG) evaluations adhering to standardized diagnostic protocols. This multimodal assessment was designed to (1) verify OSA through quantitative analysis of respiratory events (apnea-hypopnea index ≥ 5 events/hour) and oxygen desaturation patterns, and (2) systematically screen for comorbid sleep disorders. By integrating objective PSG data with structured clinical symptom inventories, conditions including narcolepsy and restless legs syndrome were methodologically excluded, thereby enhancing diagnostic reliability and supporting personalized therapeutic strategy formulation.

**Fig. 1:**
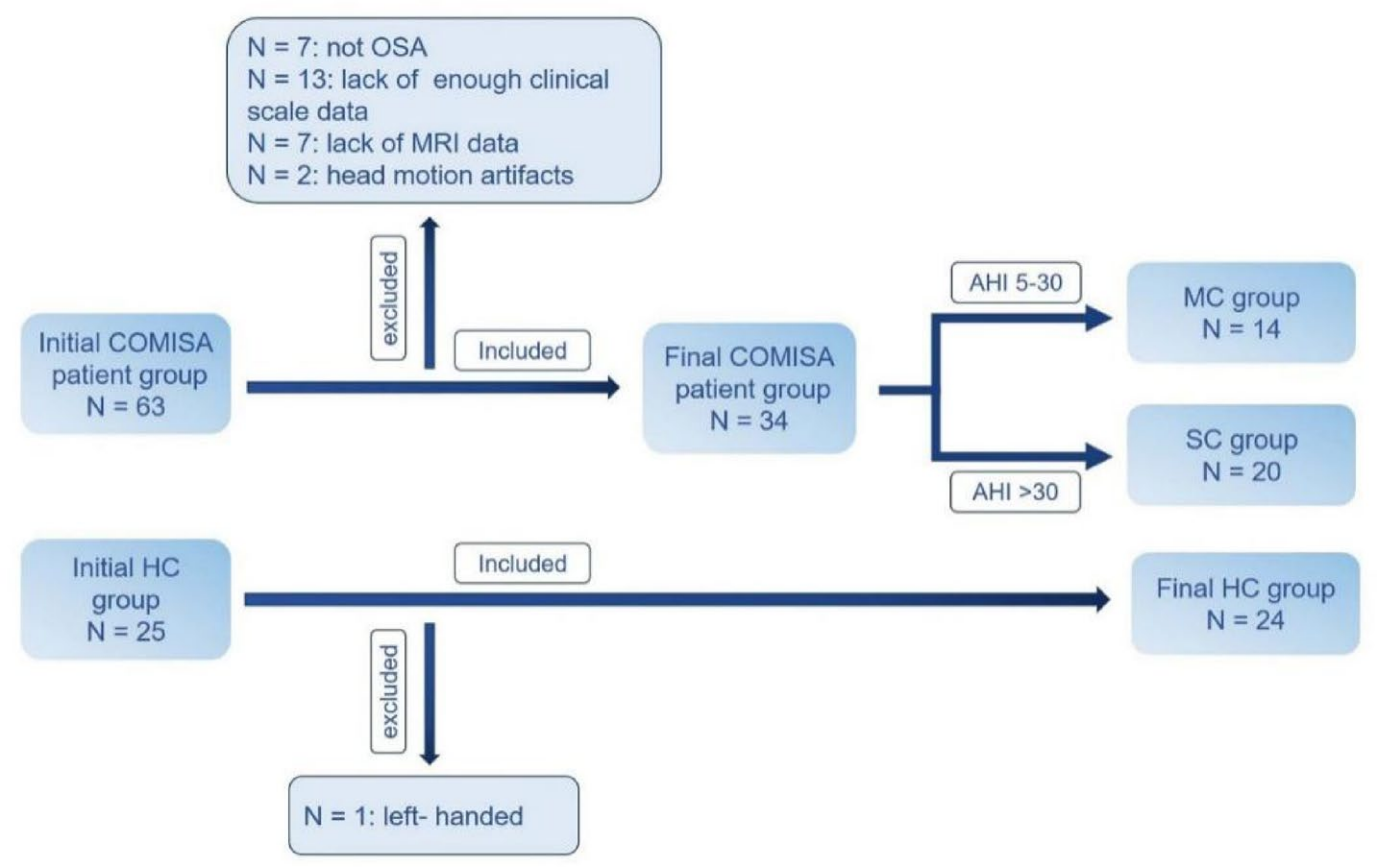
Flowchart showing the initial participant population and excluded participants. HC, Healthy control; COMISA, comorbid insomnia and sleep apnea; MC, mild and moderate COMISA; SC, severe COMISA.

Among the initial 63 participants, seven were eliminated for not satisfying the OSA diagnosis criteria, 13 were excluded because they lacked enough clinical scale data, and seven were excluded because they did not receive MRI data (Fig. 1). When two more participants were excluded due to head motion artifacts on MRI images, 34 patients (30 male and four females; age: 41.91 ± 9.15 years) were included into COMISA group. The COMISA group was further classified into mild and moderate COMISA (MC) (AHI 5–30; 14 males and for females; age: 45.21 ± 8.26 years) or severe COMISA (SC) (AHI > 30; 20 males; age: 39.60 ± 9.22 years) groups according to OSA severity.

In the HC group, 25 participants were recruited from advertisements and onsite introductions for normal physical examination. Patients below were enrolled: (1) right-handed, and (2) aged 18–60 years. Patients below were excluded: (1) those with previous or current neurological disorders or specific somatic disorders; (2) patients with current or past mental diseases themselves, or those with first-degree relatives with mental or hereditary neurological diseases; (3) those with MRI contraindications; and (4) those with excess head movement during MRI examinations. One participant was removed because they were left-handed, and 24 participants were finally included in the HC group.

### Clinical measurement

For assessing the degree of sleep apnea, sleep quality, emotion, and cognition of COMISA patients, all patients underwent the following related measurements: apnea-hypopnea index (AHI), total recorded time spent below 90% oxygen saturation (TS90%), and lowest oxygen saturation (LSaO_2_); Pittsburgh Sleep Quality Index (PSQI), Epworth Sleepiness Scale (ESS), and Insomnia Severity Index (ISI); Hamilton Depression Scale (HAMD) and Hamilton Anxiety Scale (HAMA); Montreal Cognitive Assessment (MoCA), Mini-Mental State Examination (MMSE).

### MRI data acquisition and processing

All MRI data were obtained from Xinjiang Uygur Autonomous Region Provincial General Hospital, China, and an MRI system at Phillips 3.0-T (Amsterdam, Netherlands) was used. The following MRI parameters were collected from 3D T1-weighted images: repetition time (TR) = 7.9 ms, echo time (TE) = 3.6 ms; flip angle (FA) = 8°; field of view (FOV) = 256 × 256 mm^2^; voxel size = 1.0 × 1.0 × 1.0 mm^3^; matrix size = 256 × 256; and 185 slices with no gaps covering the whole brain.

Cortical analysis was conducted with SBM with Computational Anatomy Toolbox (CAT12) in the SPM12 software. In this SBM analysis, one algorithm was used to conduct central surface and automatic CT estimates. CT estimation was conducted using projection-based methodology to determine the distance from inner (white-gray matter boundary) to the outer (gray matter-cerebrospinal fluid boundary) cortical surfaces^25^. CT was computed based on the pipeline presented in CAT12 manual under default settings, as suggested by the CAT12 manual. The 12-mm FWHM Gaussian kernel was employed to smooth the resampled CT surface data.

### Statistical analysis

All data were statistically analyzed using SPSS 26.0 (IBM, Armonk, NY, USA) and SPM12. To investigate the CT alterations in COMISA group compared to the HCs group, we performed two-sample *t*-tests on the voxel-wise whole-brain CT maps, with age, education, and BMI used as continuous covariates, and sex as a categorical covariate, in the general linear model. In line with voxel-level threshold of *p* < 0.001, *p* < 0.05 indicated statistical significance (FWE corrected at the cluster level). To investigate the effect of COMISA severity on CT, we conducted analysis of variance (ANOVA) on CT maps of MC, SC, and HC groups, covarying for the variables noted above. Significantly different brain regions identified through small-volume corrections in brain regions between COMISA and HC groups were then used to predict prior abnormalities across different levels of COMISA of varying severity. All data were of statistical significance at *p* < 0.05 (FWE corrected at a cluster level, according to the voxel-level threshold at *p* < 0.001). Given the substantial demographic imbalances across groups—particularly the all-male composition of the SC group—we supplemented our primary covariate-adjusted analyses with a male-only sensitivity comparisons and report effect sizes for all comparisons.

The CT values were extracted from the abnormal brain regions. To leverage these above-mentioned covariates, we conducted a partial correlation analysis for analyzing the relations of CT changes with the clinical factors of COMISA, MC, and SC groups. Clinical variables included the AHI, TS90%, LSaO_2_, PSQI, ESS, ISI, HAMD, HAMA, MoCA, and MMSE scores.

## Results

### Demographic and clinical measurements

Table 1 displays demographic and clinical data from HC, MC, and SC groups. The male patient proportions in MC (71%) and SC groups (100%) were greater, which met expectations due to the high sleep apnea rate in males. The HC group had a considerably lower age than MC and SC groups. The results of the Kruskal-Wallis *H* test showed that BMI and education were significantly different between three groups. As revealed by Mann–Whitney *U* test and two-sample *t*-test, SC group had higher AHI and TS90% scores than MC group. Regarding insomnia symptoms, the mean ISI score was 9.67 ± 5.07 in the MC group and 10.76 ± 4.09 in the SC group, with no significant difference between the two subgroups (*p* = 0.274). Other sleep-related measures (PSQI, ESS) also did not differ significantly between MC and SC groups (*p* > 0.05; Table 1).

**Table 1:** Clinical characteristics of HC, MC, and SC groups.

Variables	COMISA		HC AHI < 5	Statistics	*P* value
	MC	SC			
	5 ≤ AHI ≤ 30	AHI > 30			
Sex(F/M)	4/10	0/20	10/14	10.541	0.005
Age (years)	45.21 ± 8.26^a^	39.60 ± 9.22^b^	34.03 ± 9.00	16.092	< 0.001
Education level (years)	13.79 ± 1.53^a^	13.40 ± 2.28^b^	16.18 ± 3.46	26.33	< 0.001
BMI	26.72 ± 3.01^ac^	31.22 ± 5.29^bc^	24.83 ± 5.18	29.676	< 0.001
AHI (events/h)*	14.91 ± 7.52	60.97 ± 22.57	–	− 8.478	< 0.001
TS90% (min)*	27.11 ± 55.91	130.66 ± 116.24	–	− 3.78	< 0.001
LSaO_2_ (%)*	79.93 ± 8.09	72.45 ± 8.46	–	− 2.719	0.007
PSQI	8.08 ± 4.03	7.65 ± 3.20	–	0.451	0.656
ESS	7.50 ± 3.34	8.88 ± 4.21	–	− 1.064	0.296
ISI	9.67 ± 5.07	10.76 ± 4.09	–	− 1.095	0.274
HAMD	5.33 ± 5.02	3.35 ± 1.97	–	− 0.571	0.568
HAMA	5.67 ± 4.68	5.53 ± 2.45	–	− 0.207	0.839
MoCA	29.29 ± 2.05	29.24 ± 3.11	–	− 0.489	0.625
MMSE	29.25 ± 1.48	29.71 ± 0.69	–	− 0.82	0.412

HC, Healthy control; COMISA, comorbid insomnia and sleep apnea; MC, mild and moderate COMISA; SC, severe COMISA. ^a^Post hoc test results with Bonferroni correction showing significant differences between HC and MC groups. ^b^Post hoc test results with Bonferroni correction showing significant differences between HC and SC groups. ^c^Post hoc test results with Bonferroni correction showing significant differences between MC and SC groups. ^*^Two-sample t-test or Mann-Whitney U test results showing significant differences between MC and SC groups. / Untested clinical related score scale.

### SBM results for between-group voxel-wise cortical thickness map comparisons

**Fig. 2:**
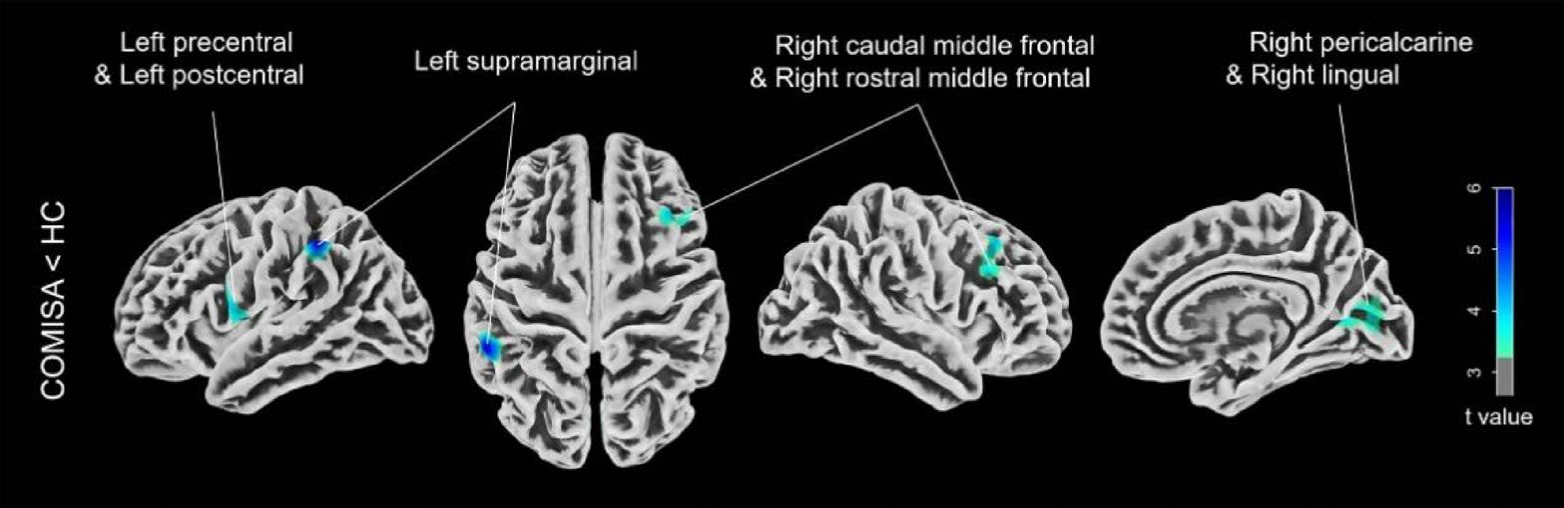
Brain regions showing decreased cortical thickness in the COMISA group compared to the HC groups. COMISA, Comorbid insomnia and sleep apnea; HC, healthy control. Color bar denotes t-statistics within the clusters that show reduced cortical thickness in patients with COMISA.

The voxel-wise whole-brain analysis revealed no significant increases in cortical thickness between groups; however, four clusters in the COMISA group showed significantly reduced cortical thickness compared to the HC group (Table 2; Fig. 2). Those four clusters of lower CT primarily located in left supramarginal gyrus, left precentral and left postcentral gyri, right pericalcarine and right lingual gyri, and right rostral and caudal middle frontal gyri.

**Table 2:** Comparison of brain regions with decreased cortical thickness in COMISA and HC groups (FWE correction, *p* < 0.001).

Group	Clusters	Cluster size	Brain regions	Peak MNI coordinate			Peak t value	Cohen’s d
				x	y	z		
COMISA < HC	Cluster 1	111	Left supramarginal	− 55	− 43	47	5.46	1.46
	Cluster 2	94	Left precentral	− 60	3	11	4.07	1.09
			Left postcentral	− 59	7	22	3.62	0.97
	Cluster 3	205	Right pericalcarine	11	− 80	4	3.81	1.02
			Right lingual	6	− 65	7	3.85	1.03
	Cluster 4	167	Right caudal middle frontal	38	26	46	4.07	1.09
			Right rostral middle frontal	45	21	32	3.83	1.02

HC, Healthy control; COMISA, comorbid insomnia and sleep apnea; MNI, Montreal Neurological Institute; cluster size: in mm^3^; all Cohen’s d values indicate large to very large effect sizes (> 0.8).

**Fig. 3:**
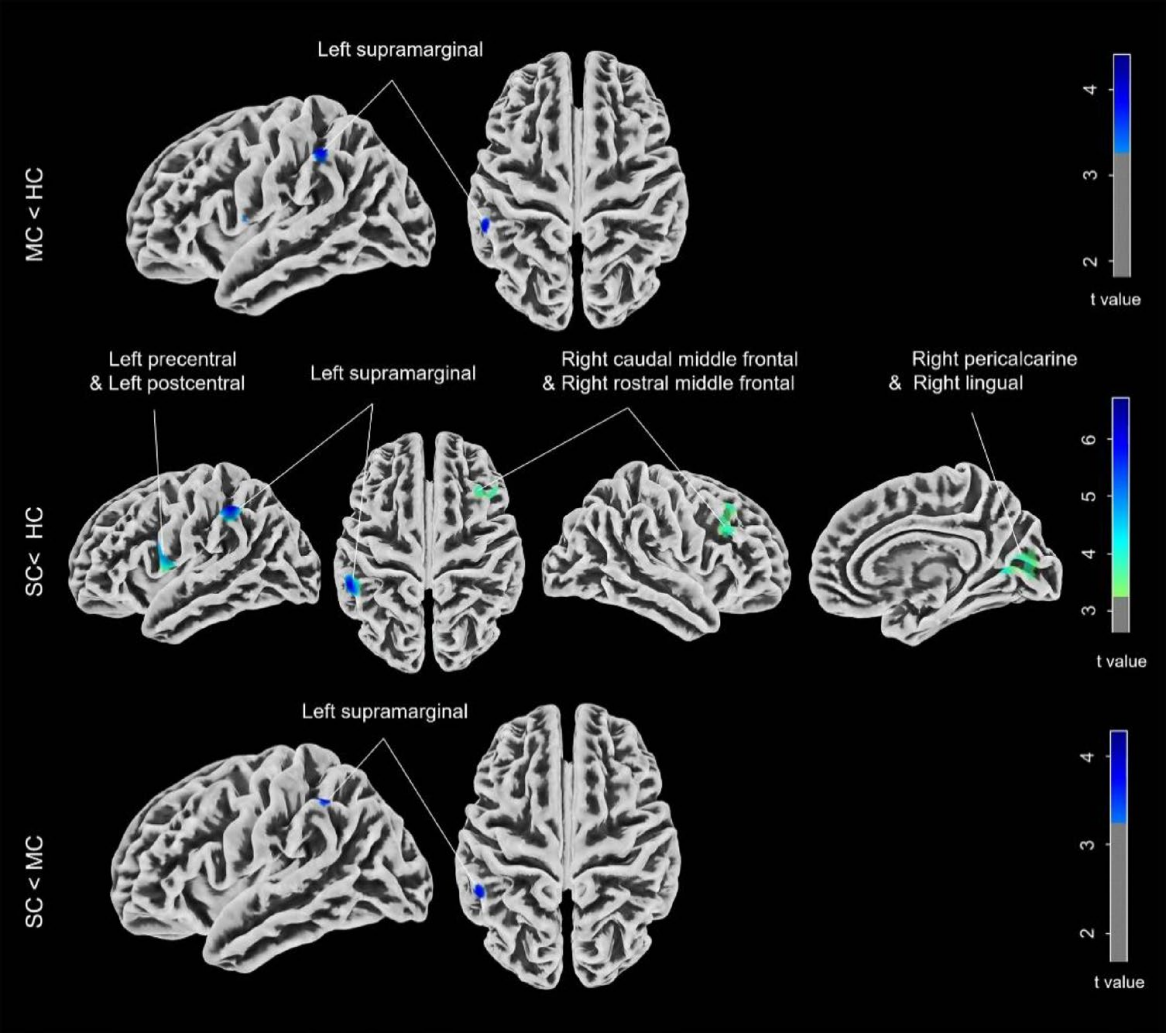
Expanded decreased cortical thickness with the severity of patients with COMISA. COMISA, Comorbid insomnia and sleep apnea; HC, healthy control; MC, mild and moderate COMISA; SC, severe COMISA. Color bar denotes t-value.

To explore whether varying OSA severity within COMISA affected the CT, we conducted ANOVA plus post hoc analysis of individual CT maps from MC, SC, and HC groups. Our results of ANOVA also showed prominent group differences in CT in the left supramarginal gyrus, left precentral and left postcentral gyri, right pericalcarine gyrus and right lingual gyrus, and right rostral and caudal middle frontal gyri. Post hoc tests following ANOVA showed that CT in left supramarginal gyrus of MC group significantly decreased relative to HC group (Table 3; Fig. 3). Moreover, SC group showed more extensive CT reduction in the left supramarginal gyrus based on cluster size, along with additional CT reductions in left precentral and left postcentral gyri, right pericalcarine and right lingual gyri, and right rostral and caudal middle frontal gyri, than the HC group. Finally, CT in left supramarginal gyrus of SC group decreased compared with MC group.

**Table 3:** Comparison of brain regions with decreased cortical thickness in MC, SC, and HC groups (FWE correction, *p* < 0.001).

Group	Clusters	Cluster size	Brain regions	Peak MNI coordinate			Peak t value	Cohen’s d
				x	y	z		
MC < HC	Cluster 1	32	Left supramarginal	− 57	− 40	45	4.01	1.34
SC < HC	Cluster 1	111	Left supramarginal	− 55	− 43	47	6.12	1.89
	Cluster 2	94	Left precentral	− 58	7	25	5.12	1.58
			Left postcentral	− 60	4	13	4.96	1.53
	Cluster 3	200	Right pericalcarine	11	− 80	4	4.05	1.25
			Right lingual	6	− 62	5	4.14	1.28
	Cluster 4	161	Right caudal middle frontal	37	26	48	3.99	1.23
			Right rostral middle frontal	46	25	28	3.97	1.22
SC < MC	Cluster 1	16	Left supramarginal	− 53	− 47	47	3.90	1.38

HC, Healthy control; COMISA, comorbid insomnia and sleep apnea; MC, mild and moderate COMISA; SC, severe COMISA; MNI, Montreal Neurological Institute; cluster size: in mm^3^; all Cohen’s d values indicate large to very large effect sizes (> 0.8).

### SBM results of male-only sensitivity comparisons

The male‑only sensitivity analysis revealed a largely similar pattern of cortical thinning as the original analysis, with the COMISA group showing significantly decreased cortical thickness in the left supramarginal gyrus, left precentral gyrus, left caudal and rostral middle frontal gyri, left pars triangularis, left superior frontal gyrus, right pericalcarine gyrus and right lingual gyrus, as well as right caudal and rostral middle frontal gyri (Supplementary Table S3, Supplementary Fig. S2). Compared to the original findings (Table 2), three additional clusters were identified in the male‑only subsample: left caudal and rostral middle frontal gyri, left pars triangularis gyrus, left superior frontal gyrus). Post hoc tests yield results largely consist with the original result, with the exception that no significant difference was observed between the SC and MC groups (Supplementary Table S3). These sensitivity analyses support the robustness of the main findings.

### Correlation analysis

**Fig. 4:**
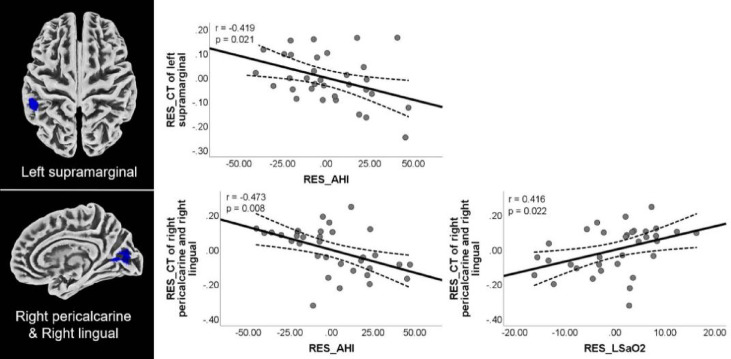
Partial correlations between cortical thickness and clinical variables in the COMISA group. The cortical thickness of left supramarginal gyrus was negatively correlated with the AHI score; The cortical thickness of right pericalcarine and right lingual gyri was negatively correlated with the AHI score, positively correlated with the LSaO_2_. COMISA, Comorbid insomnia and sleep apnea; AHI, apnea-hypopnea index; LSaO_2_, lowest oxygen saturation.

The results of partial correlation analysis showed a negative relation of CT in left supramarginal gyrus with the AHI of COMISA group (*r* = − 0.419, *p* = 0.021; Fig. 4). CT of the right pericalcarine and right lingual gyri were negatively related to the AHI but positively related to the LSaO_2_ score of COMISA group (*r* = − 0.473, *p* = 0.008 and *r* = 0.416, *p* = 0.022, separately; Fig. 4). However, mean CT values of abnormal brain regions did not show significant relation to clinical variable scores in both MC and SC groups.

## Discussion

Our findings showed that the patients with COMISA had CT reductions in four clusters, namely, left supramarginal gyrus, left precentral and left postcentral gyri, right pericalcarine and right lingual gyri, and right rostral and caudal middle frontal gyri. These brain areas are related to respiratory and sleep disorders^5,26,27^. A reduction in CT of left supramarginal gyrus could be found in MC group, whereas four clusters were found in the SC group. We also found expanded reduced CT in the left supramarginal gyrus according to the cluster size in SC group.

The partial correlation analysis results suggested that the CT in left supramarginal gyrus was significantly and negatively related to AHI of the COMISA group. Additionally, the CT of the right pericalcarine and right lingual gyri were significantly and negatively related to the AHI score but positively related to the LSaO_2_ score in the COMISA group. This study provided complementary and empirical evidence that varying OSA severity within COMISA is related to greater cortical thinning in several different brain areas and that the apnea caused by COMISA and changes in blood oxygen levels may contribute to the reduction in CT in patients with COMISA. Compared to our previous GMV findings in the same cohort^20^, the current CT results reveal different regional patterns (sensorimotor, parietal, occipital, frontal, temporal, fusiform), distinct correlation profiles (AHI, LSaO₂, TS90%, MMSE, HAMD), and a stepwise severity effect. Our findings align with previous studies demonstrating that GMV and CT reflect distinct biological information^21,22^, as evidenced by the divergent regional patterns and correlation profiles observed between the current CT analysis and our prior GMV study in the same cohort. These dissociations suggest that cortical thickness captures complementary aspects of COMISA-related brain pathology that are not reducible to gray matter volume changes. However, these correlational findings do not imply causation. Other mechanisms associated with OSA and insomnia, such as sleep fragmentation, neuroinflammation, or oxidative stress, could also contribute to the observed cortical thinning and should be investigated in future studies.

We found that the left supramarginal gyrus exhibited cortical thinning in COMISA patients compared to that in HCs. The supramarginal gyrus, an important component of the inferior parietal lobule, is involved in respiratory processes and functions^26^. OSA patients commonly experience respiratory control dysfunction, leading to sleep fragmentation characterized by frequent awakenings due to sleep apnea. Respiratory control disorder and persistent sleep-deprived state is hypothesized to be associated with morphological changes of brain structure^28,29^, thereby may facilitating the complex pathophysiology of OSA. Joo et al.^5^ found that cortical thinning can be located in the inferior parietal lobule, with more respiratory arousals being associated with cortical thinning in inferior parietal lobule and anterior cingulate of OSA patients. Chokesuwattanaskul et al.^17^ found that the desaturation magnitude and frequency in OSA patients were related to the decrease in CT in the right inferior parietal gyrus. These results suggest that sleep fragmentation and accompanying oxygen desaturation contribute to morphometric alterations in the inferior parietal lobule or supramarginal gyrus. As shown in previous studies, we found a reduction in CT of the left supramarginal gyrus in COMISA patients, which was negatively correlated with the AHI. As suggested by our correlation analysis, the increased apnea-hypopnea events contribute to greater thinning of the cortical tissue in the left supramarginal gyrus. Furthermore, CT in the left supramarginal gyrus of MC group decreased compared with HC group, and CT in the left supramarginal gyrus decreased to a greater extent based on the cluster size in SC group. Additionally, CT of the left supramarginal gyrus in SC group decreased relative to MC group. This might be due to greater severity of apnea and intermittent hypoxia in SC relative to MC groups, leading to thinning of left supramarginal gyrus in SC group.

Our results indicated that COMISA patients showed considerably lower CT values in both left precentral and postcentral gyri than HCs. Besides, both precentral and postcentral gyri belong to the sensorimotor cortex^30^. Some studies have found precentral and postcentral gyrus thinning in OSA and ID patients^5,18,31^. A study^5^ reported obvious cortical thinning in both bilateral precentral and postcentral gyri of OSA patients compared to that of HCs, which was related to the arousal index, respiratory arousal, and maximum duration of apnea. Patients with persistent insomnia showed cortical thinning in the precentral cortex compared to good sleepers^18^. Some studies have also shown that volume deficits in gray matter in the precentral gyrus in insomnia patients are associated with sleep disorders^32,33^. Based on these findings, sleep disturbance and hypoventilation are related to morphometric alterations in the precentral gyrus. Similar to the findings of previous studies, we found cortical thinning of both left precentral and postcentral gyri in patients in the COMISA group and the SC group. However, cortical thinning in both left precentral and postcentral gyri was not observed in the MC group. This occurred probably because a decrease in CT occurs in the severe stage of COMISA, potentially due to severe apnea and lower blood oxygen saturation levels, which are more prevalent in the SC group.

The CT values of the right pericalcarine and lingual gyri located in the occipital lobe remarkably decreased in COMISA patients relative to HCs. The lingual gyrus of the occipital lobe, situated between the calcarine sulcus and the posterior segment of the collateral sulcus and extending to the tentorial surface of the temporal lobe, where it joins the hippocampus^34^, is part of the visual center^35^. A study showed that hypoxic exposure exerts long-term effects on the visual cortex^36^. These findings suggested that the cortex of the right pericalcarine and lingual gyri was thinner in COMISA patients than in HCs. CT in the above regions was negatively related to the AHI but positively related to LSaO_2_, suggesting that an increase in the apnea-hypopnea index and a decrease in minimum nighttime oxygen saturation are associated with a thinner cortex in the right pericalcarine and lingual gyri. Moreover, cortical thinning of both left precentral and postcentral gyri was observed in SC relative to HC groups; however, these changes were not recorded in the MC group. This finding indicated that greater cortical thinning in more severe cases of COMISA is consistent with the possibility that greater hypoxia severity contributes to cortical thinning. However, relevant studies on the pericalcarine region are limited. More research is needed for elucidating the cortical thinning mechanism in the pericalcarine gyrus in COMISA patients, particularly by analyzing the complicated and different effects of factors such as respiration and sleep on the structure of the brain.

The middle frontal gyrus (MFG), composed of rostral and caudal regions^37^, is a part of the frontal-parietal control network and is associated with visuospatial planning, cognitive control, attention, and memory^38^. Our findings showed that CT in the right caudal and rostral MFG was lower in the COMISA group than in HCs. Zhou et al.^27^ reported that the regional homogeneity (ReHo) in the MFG in moderate-to-severe OSA patients decreased. They also found that abnormal ReHo values in the MFG were negatively correlated with the AHI and oxygen desaturation index, implying that intermittent hypoxemia may affect the functions of the prefrontal lobe. OSA is characterized by chronic intermittent hypoxemia and fragmentation of sleep, which can lead to neuronal cell impairment. Severe hypoxia may decrease cell metabolism^39^ and increase the death of cerebral cortex neurons^40,41^. This direct loss of cells or a decrease in their volume can manifest as cortical thinning^42^. Therefore, cortical thinning of the right caudal and rostral MFG in COMISA patients may be related to hypoxia. This speculation was further supported by our finding that cortical thinning of the right caudal and rostral MFG was observed in SC rather than MC group.

The male-only sensitivity analysis yielded results largely mirroring those of the original analysis: the COMISA group continued to exhibit significant cortical thinning in the originally identified clusters, along with three additional clusters. However, in contrast to the original findings, the male-only analysis revealed no significant cluster-level differences between the severe COMISA (SC) and moderate COMISA (MC) groups. These patterns suggest that while the overall COMISA–healthy control (HC) differences are robust to sex stratification, the specificity of cortical thinning across COMISA severity levels may be partially influenced by demographic imbalances, including sex distribution. Consequently, the generalizability of our findings to female patients with severe COMISA remains limited, and certain observed differences—particularly between the SC and MC groups—should be interpreted with caution. Future studies with larger, well-matched samples (balanced by sex, age, and body mass index) are essential to confirm whether the cortical thinning patterns reported here can be specifically attributed to COMISA severity rather than to demographic confounds.

This study had certain limitations. First, we only orally asked participants questions relevant to the study and did not obtain objective measurements associated with respiration, sleep, emotion, and cognition in HC group. This may result in subjectivity, inaccuracies, and insufficient standardization of the collected data. Moreover, the absence of systematic assessments for sleep, mood, and cognition in the HC group limits our ability to confirm their ‘healthy’ status beyond self-report, and future studies should include such evaluations. Thus, future studies must measure the relevant parameters from participants. Second, a limitation of this study is the inability to ascertain whether the observed changes in COMISA patients are due exclusively to OSA, exclusively to ID, or an interaction of both, given the absence of control groups for OSA-only and ID-only patients. To accurately delineate the relationship between OSA and ID, future investigations should incorporate these specific control groups. Third, the COMISA severity was not determined due to the absence of a standard or scale for assessing its severity. Consequently, patients were categorized into COMISA subgroups according to the severity of OSA. Our findings therefore specifically reflect the impact of OSA severity within a COMISA cohort, not a true composite measure of COMISA severity. Further studies are needed to establish standards to measure the severity of COMISA. Forth, the total sample size of 34 COMISA patients, particularly after splitting into subgroups (MC: *n* = 14, SC: *n* = 20), is relatively small for whole brain voxel wise analyses and partial correlation studies. This increases the risk of type II errors (false negatives) and may lead to unstable effect estimates. Therefore, our findings should be considered exploratory and require validation in larger independent cohorts. Moreover, our correlational analyses cannot disentangle the effects of hypoxia from other potential contributors such as sleep fragmentation or inflammation.

## Conclusion

To summarize, we found a greater cortical thinning from the left supramarginal gyrus to brain regions located in the sensorimotor-, frontal- and visual-related cortices with increasing OSA severity within COMISA. CT reduction in the supramarginal gyrus highlights early brain structural alterations in patients with COMISA. The significant correlations between CT and respiration measures (AHI and LSaO_2_) are consistent with a possible contribution of respiratory impairment (e.g., intermittent hypoxia) to cortical thinning in patients with COMISA.

## Supplementary Information

Below is the link to the electronic supplementary material.

Supplementary Material 1

Supplementary Material 2

## Data Availability

The datasets generated during and/or analyzed during the current study are available from the corresponding author upon reasonable request.
