# Supplementary material for: Cortical thinning with increasing OSA severity stages in adult comorbid insomnia and sleep apnea and its relevance for respiration measures
Source: Sci Rep. 2026 May 28;16:24403. doi: 10.1038/s41598-026-55300-7 (PMC13448248; doi:10.1038/s41598-026-55300-7)
Supplement: Supplementary file 1 [file 41598_2026_55300_MOESM1_ESM.docx]

Fig. S2. Brain regions showing decreased cortical thickness in COMISA, MC, and SC groups compared to HC groups in male-only sensitivity analysis. Abbreviations: COMISA, comorbid insomnia and sleep apnea; HC, healthy control; MC, mild and moderate COMISA; SC, severe COMISA. Color bar denotes t-statistics within the clusters showing reduced cortical thickness in patients with COMISA across different subsample definitions.
