# Supplementary material for: Cortical thinning with increasing OSA severity stages in adult comorbid insomnia and sleep apnea and its relevance for respiration measures
Source: Sci Rep. 2026 May 28;16:24403. doi: 10.1038/s41598-026-55300-7 (PMC13448248; doi:10.1038/s41598-026-55300-7)
Supplement: Supplementary file 2 [file 41598_2026_55300_MOESM2_ESM.docx]

| Table S3. Sensitivity analysis: comparison of brain regions with decreased cortical thickness between groups in the male‑only subsample (FWE correction, p < 0.001). | | | | | | | | |
| --- | --- | --- | --- | --- | --- | --- | --- | --- |
|  | Group | clusters | cluster size | Brain regions | Peak MNI coordinate | | | peak t value |
|  |  |  |  |  | x | y | z |  |
| Male‑only | COMISA<HC | cluster 1 | 130 | Left supramarginal | -55 | -43 | 47 | 6.17 |
|  |  | cluster 2 | 269 | Left precentral | -47 | 4 | 19 | 4.50 |
|  |  | cluster 3 | 125 | Left caudal middle frontal | -42 | 13 | 51 | 4.77 |
|  |  | cluster 4 | 89 | Left rostral middle frontal | -35 | 51 | 7 | 4.25 |
|  |  | cluster 5 | 89 | Left pars triangularis | -50 | 27 | 4 | 4.14 |
|  |  | cluster 6 | 139 | Left superior frontal | -18 | 6 | 67 | 3.95 |
|  |  | cluster 7 | 256 | Right pericalcarine | 8 | -64 | 6 | 5.05 |
|  |  |  |  | Right lingual | 13 | -79 | 5 | 4.15 |
|  |  | cluster 8 | 514 | Right caudal middle frontal | 41 | 19 | 33 | 5.05 |
|  |  |  |  | Right rostral middle frontal | 42 | 21 | 20 | 4.20 |
|  | MC<HC | cluster 1 | 39 | Left supramarginal | -55 | -43 | 47 | 4.79 |
|  |  | cluster 2 | 90 | Right caudal middle frontal | 39 | 18 | 32 | 4.78 |
|  |  |  |  | Right rostral middle frontal | 38 | 26 | 46 | 3.48 |
|  |  | cluster 3 | 37 | Right lingual | 11 | -64 | 6 | 4.52 |
|  | SC<HC | cluster 1 | 111 | Left supramarginal | -54 | -44 | 47 | 6.52 |
|  |  | cluster 2 | 93 | Left precentral | -58 | 7 | 25 | 4.95 |
|  |  | cluster 3 | 163 | Right caudal middle frontal | 41 | 22 | 36 | 4.73 |
|  |  |  |  | Right rostral middle frontal | 37 | 27 | 47 | 3.99 |
|  |  | cluster 4 | 152 | Right pericalcarine | 6 | -64 | 6 | 4.56 |
|  |  |  |  | Right lingual | 9 | -72 | 14 | 4.17 |
|  | SC<MC | none | | | | | | |
